# Osteopontin and Cancer: Insights into Its Role in Drug Resistance

**DOI:** 10.3390/biomedicines11010197

**Published:** 2023-01-12

**Authors:** Chengcheng Hao, Jane Lane, Wen G. Jiang

**Affiliations:** 1 Department of Oncology, Beijing Shijitan Hospital, Capital Medical University, Beijing 100038, China; 2 Cardiff China Medical Research Collaborative, Division of Cancer and Genetics, Cardiff University School of Medicine, Heath Park, Cardiff CF14 4XN, UK

**Keywords:** osteopontin, cancer, drug resistance, molecular mechanism

## Abstract

Cancer is one of the leading causes of mortality worldwide. Currently, drug resistance is the main obstacle in cancer treatments with the underlying mechanisms of drug resistance yet to be fully understood. Osteopontin (OPN) is a member of the integrin binding glycophosphoprotein family that is overexpressed in several tumour types. It is involved in drug transport, apoptosis, stemness, energy metabolism, and autophagy, which may contribute to drug resistance. Thus, understanding the role of OPN in cancer drug resistance could be important. This review describes the OPN-based mechanisms that might contribute to cancer drug resistance, demonstrating that OPN may be a viable target for cancer therapy to reduce drug resistance in sensitive tumours.

## 1. Introduction

Despite the significant advancements in treatment throughout the decades, cancer remains one of the leading causes of human death globally, with 10 million deaths in 2020 [1]. Currently, drug therapy remains one of the well-established and promising methods of cancer healing in clinical practice, particularly for patients after tumour removal. A number of anti-cancer drugs, including chemotherapy drugs, molecular targeted drugs and immunotherapy drugs, have been developed and have somewhat improved early clinical efficacy [2]. However, the occurrence of drug resistance after long-term administration often leads to cancer treatment ineffectiveness and eventual cancer-related mortality [3,4]. According to statistical reports, nearly 90% of cancer deaths are directly or indirectly attributed to drug resistance [5]. Therefore, cancer drug resistance in response to treatment remains one of the greatest impediments for achieving relapse-free survival outcomes in patients. A better understanding of the potential molecular mechanisms that evade therapeutic response, and the development of precise strategies to combat resistance are urgent and significant medical needs in the treatment of human cancers.

**Figure 1:**
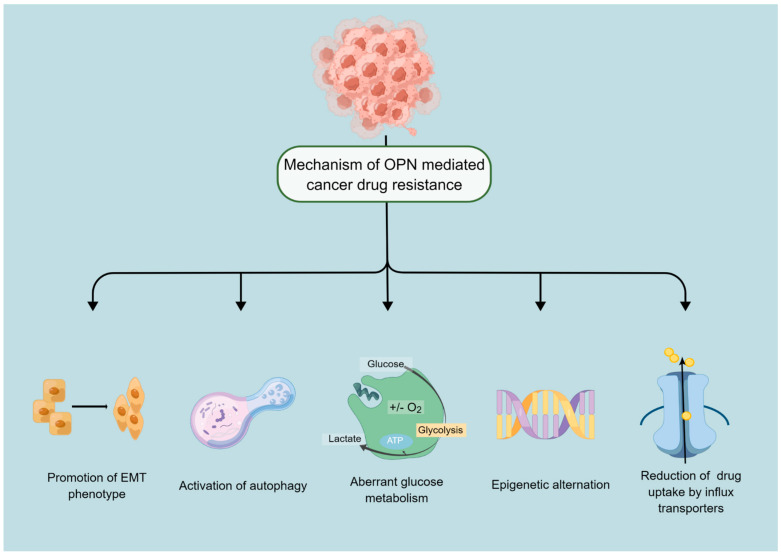
Mechanisms of anticancer drug resistance mediated by OPN. Cancer cells can survive and relapse through different mechanisms mediated by OPN, which are depicted in the above figure. Mechanisms mainly include EMT, autophagy, energy metabolism, epigenetic regulation, and drug efflux. By Figdraw (www.figdraw.com, accessed on 2 January 2023).

**Figure 2:**
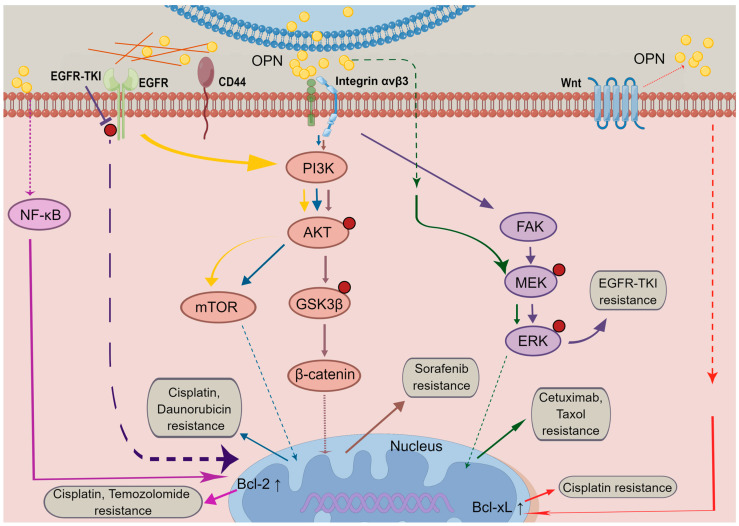
OPN-induced signalling pathways in cancer drug resistance. The OPN expression is regulated by autocrine and paracrine pathways. Upon OPN binding to integrin or CD44 receptors, especially by integrin αvβ3, PI3K/Akt signalling, MAPK signalling, and EGFR signalling are triggered either directly or indirectly, leading to cancer cell survival and therapeutic resistance. By Figdraw (www.figdraw.com, accessed on 2 January 2023).

OPN, also known as SPP1 (secreted phosphoprotein 1), a small integrin-binding glycophosphoprotein has been implicated in many pathophysiological processes including tumourigenesis [6,7]. During the past few decades, extensive research has demonstrated OPN is frequently expressed at increased levels in many types of cancer, which contributes to malignant phenotype and poor prognosis, and may become a useful biomarker to monitor cancer progression and a significant predictor of survival [8,9,10,11,12]. Recent findings further showed that aberrant expression of OPN has been positively correlated with poor treatment response in different types of cancer, and modulating the expression of OPN may reverse drug resistance. Accordingly, in the present review, recent approaches regarding the functions of OPN and its splice variants, to promote tumour resistance to drugs in both clinical and experimental studies are summarised. The potential mechanisms by which OPN is involved in tumour resistance (Figure 1) and associated signalling pathways (Figure 2) are also highlighted to provide reference for clinical treatment.

## 2. OPN Expression for the Evaluation of Response to Cancer Treatment

Recently, there has been ongoing clinical research regarding the function of OPN in cancer drug resistance. Accumulative evidence suggests that OPN correlates with clinical resistance to chemotherapy and radiotherapy, in a variety of human tumours (Table 1). 

### 2.1. OPN Expression and Response to Radiation Therapies

It has been shown that OPN expression was associated with chemoradiotherapy sensitivity in cervical cancer patients. Compared to chemoradiotherapy-sensitive patients, those with chemoradiotherapy-resistance had higher overexpression rates of OPN (89% VS 58%, *p* = 0.009) [13]. In addition, a significantly higher percentage of patients with positive OPN expression in the radiation-resistant group, compared with those in the radiation-sensitive group (*p* < 0.001), documented that high OPN expression was a potential indicator for radiation resistance, in locally advanced cervical squamous cell carcinoma (LACSCC) [14].

### 2.2. OPN Expression in Tumours and Response to Chemotherapies

There has been some compelling evidence that expression levels of OPN are closely connected with patient response to drug treatment. It has been shown that OPN expression levels were significantly associated with the efficacy of platinum-based treatment in patients with advanced non-small cell lung cancer (NSCLC) (*p* = 0.038) [15]. Moreover, multivariate analysis indicated that OPN expression level may be an independent factor for predicting the tumour response to first-line platinum-based chemotherapy (HR = 2.326, 95% CI:1.721–2.616, *p* = 0.005). Another investigation demonstrated that patients with a low expression of OPN (19/27, 70%) were more likely to respond to cisplatin-based induction chemotherapy than those with a high expression of OPN (33/94, 35%), in locally advanced oral squamous cell carcinoma (OSCC) (*p* = 0.002) [16]. However, no significant relationship was observed between the expression levels of OPN and response to taxane-based therapeutic regimen (TBR), in patients with prostate cancer (PC) (*p* = 0.622) [17].

### 2.3. Serum Levels of OPN and Patients’ Responses to Treatment

There are investigations which suggest that baseline OPN in serum can be used as a biomarker for the evaluation of response to cancer treatment. As shown in Table 2, high pretreatment serum OPN was significantly correlated with poor chemoradiotherapy response, in cervical cancer patients: 74.01 ± 27.95 ng/mL in patients with a complete response (CR) and 116.98 ± 10.36 ng/mL in those who achieved a partial response (PR), improvement (MR), or progressive disease (PD) (*p* < 0.001) [13]. Additionally, the prospective study in a metastatic breast cancer cohort demonstrated that pretreatment OPN levels of chemotherapy refractory patients were significantly higher than that of the chemotherapy responsive ones (49.1 ± 33.8 ng/dL, 35.5 ± 34.3 ng/dL, respectively, *p* = 0.05) [18]. Another study by Yazici et al., also demonstrated that baseline values of serum OPN increased with the worsening of response to the docetaxel, cisplatinum, and 5-fluorouracil combination (DCF) regimen, in advanced gastric cancer (GC) (110.7 ± 29.3 ng/mL in responders, and 211.9 ± 24.4 ng/mL in non-responders, respectively, *p* = 0.002) [9]. Similarly, the study using quantitative real-time reverse transcription-PCR (qRT-PCR) analyses showed that patients with high OPN gene expression had a poor response to 5-FU-based chemoradiotherapy, or showed more drug resistance in OSCC (*p* < 0.05) [19]. Different from prior research, Xu et al., suggested that, among small cell lung cancer (SCLC) patients with different response to first-line chemotherapy with etoposide and cisplatin (EP), or etoposide and carboplatin (EC), there was no significant difference in OPN level (*p* = 0.485) [20].

Therefore, these studies reveal that high OPN expression would be strongly linked with poor response, suggesting that OPN could be considered as a biomarker for cancer progression and a potential target for increasing the therapeutic efficacy in some tumours. Yet it is worth noting that the prognostic value of OPN in tissues or blood may vary due to differences in tumour type, tumour stage, therapeutic regimens or detection methods. The correlational studies require further systematisation with a view to applying this biomarker to clinical applications.

**Table 1:** Correlation between expression of OPN and treatment response in cancer patients.

Cancer Types	Study	Inclusion Year	Sample Size	Cancer Stage	Therapeutic Regimens	Test Method	Treatment Response	n, (%)	OPN Expression		*p* Value	*HR/OR (95% CI)*	*p* Value
									Low n, (%)	High n, (%)			
Cervical SCC	Feng et al. 2018 [13]	January 2015–December 2015	116	IIB-IIIB	IMRT + platinum-based chemotherapy	IHC	Sensitive (CR)	97 (84%)	41 (95%)	56 (77%)	0.009		
							Resistant (PR + MR + PD)	19 (16%)	2 (5%)	17 (23%)			
NSCLC	Huang et al. 2018 [14]	March 2010–August 2016	73	I-IV	First-line platinum-based chemotherapy	IHC	PR	27 (37%)	12 (38%)	15 (37%)	0.038	2.326 (1.721–2.616)	0.005
							SD	38 (52%)	18 (56%)	20 (49%)			
							PD	8 (11%)	2 (6%)	6 (15%)			
OSCC	Luo et al. 2015 [16]	2006–2012	121	IV	IC + CRRT	IHC	CR/PR	52 (43%)	19 (70%)	33 (35%)	0.002	0.320 (0.120–0.854)	0.023
							SD/PD	69 (57%)	8 (30%)	61 (65%)			
PC	Aksoy et al. 2017 [17]	2009–2015	30	IV	TBR	IHC	Responders	12 (40%)	1 (20%)	11 (44%)	0.622		
							Non-responders	18 (60%)	4 (80%)	14 (56%)			
LACSCC	Huang et al. 2015 [16]	January 2005–March 2012	111	Ib-Iva (FIGO)	Primary radical radiotherapy	IHC	Radiation-sensitive	85 (77%)	59 (95%)	26 (53%)	<0.001		
							Radiation-resistant	26 (23%)	3 (5%)	23 (47%)			

Abbreviations: NSCLC, non-small-cell lung cancer; OSCC, oral squamous cell carcinoma; PC, prostate cancer; LACSCC, locally advanced cervical squamous cell carcinoma; CR, complete response; MR, improvement; PR, partial response; SD, stable disease; PD, progressive disease; IC, induction chemotherapy; CCRT, concurrent chemoradiotherapy; HR, hazard ratio; OR, odds ratio; CI, confidence interval.

**Table 2:** Tumour response in patients with baseline OPN levels.

Cancer Type	Study	Inclusion Year	Sample Size	Cancer Stage	Therapeutic Regimens	Test Method	Treatment Response	n	OPN Expression	*p* Value
Cervical cancer	Feng et al. 2018 [13]	January 2015–December 2015	116	IIB-IIIB	IMRT + platinum-based chemotherapy	ELISA	Sensitive (CR)	97 (84%)	74.01 ± 27.95 (ng/mL)	<0.001
						Resistant (PR + MR + PD)	19 (16%)	116.98 ± 10.36 (ng/mL)		
SCLC	Xu et al. 2020 [20]	_	96	_	First-line chemotherapy (EP/EC)	ELISA	CR + PR	78 (81%)	71.15 ± 18.33 (ng/mL)	0.485
							SD + PD	18 (19%)	78.01 ± 13.74 (ng/mL)	
Metastatic breast cancer	Elbaiomy et al. 2020 [18]	January 2017–March 2019	115	IV	First-line chemotherapy (DC)	ELISA	CR, PR, and SD	41 (36%)	35.5 ± 34.3 (ng/dL)	0.05
							PD	73 (64%)	49.1 ± 33.8 (ng/dL)	
GC	Yazici et al. 2020 [9]	2009–2015	42	IV	Modified DCF	ELISA	Responders	_	110.7 ± 29.3 (ng/mL)	0.002
							Non-responders	_	211.9 ± 24.4 (ng/mL)	
OSCC	Nakamura et al. 2014 [19]	1999–2004	49	I-IV	5-FU-based chemoradiotherapy	qRT-PCR	Sensitive	30 (61%)	OPN overexpression in OSCC tissues with resistance to 5-FU-based chemoradiotherapy	<0.05
							Resistant	19 (39%)		

Abbreviations: SCLC, small-cell lung cancer; OSCC, oral squamous cell carcinoma; GC, gastric cancer; IMRT, intensity modulated external radiation therapy; EP, etoposide and cisplatin; EC, etoposide and carboplatin; DC, docetaxel 75 mg/m^2^+cisplatin 75mg/m^2^ or carboplatin 6 AUC (area under the curve); DCF, docetaxel 60 mg/m^2^ on 1.day, cisplatin 60 mg/m^2^ on 1.day, 5-fluorouracil 750 mg/m^2^ on days 1–4; CR, complete response; MR, improvement; PR, partial response; SD, stable disease; PD, progressive disease.

## 3. Mechanism of OPN Mediated Anticancer Drug Resistance

### 3.1. OPN Is a Potential Regulator of Autophagy

Autophagy is an intracellular self-degradation process, by which cytoplasmic components are delivered to lysosomes for degradation and recycling, which is required to maintain cellular homeostasis and quality control [21]. Dysregulated autophagy is highly prevalent in a wide range of human diseases, including cancer [22,23]. Considerable evidence has supported the observation that autophagy frequently occurs during cancer progression and in response to anti-cancer treatment [24,25,26,27]. Sustained activation of autophagy improves cytoprotection by conferring cytotoxic stress tolerance and limiting apoptosis, leading to cancer drug resistance and tumour promotion, especially during later stages of tumourigenesis [28,29,30].

Recently, OPN has been described as a key modulator of autophagy. OPN is able to stimulate autophagy directly, via integrin/CD44 and p38 mitogen-activated protein kinase (MAPK) signalling pathways, in vascular smooth muscle cells [31]. Down-regulated expression of OPN could dramatically inhibit osteogenic differentiation and mineralisation by autophagy inhibitors [32]. Moreover, in solid tumours, it has been reported that OPN induces chemoresistance and radioresistance through regulating the autophagy signalling pathways. In hepatocellular carcinoma (HCC) cells, OPN accumulation elicits autophagy via binding with its receptor integrin αvβ3 and sustaining Forkhead box (Fox)O3a stability, which further promotes tumour growth and resistance to epirubicin and cisplatin [33]. In vitro and in vivo studies on pancreatic cancer indicated that OPN promotes the expression of autophagy-related genes, including LC3-II, ATG5, and ATG7, by activating the nuclear factor κ-light-chain-enhancer of activated B cells (NF-κB) signalling pathway, leading to enhanced autophagy and resistance to gemcitabine. The OPN/NF-κB/autophagy pathway may act as an attractive target for eliminating pancreatic chemoresistance [34]. In addition, recent lines of evidence show that OPN can serve as an indicator of resistance to radiotherapy. Elevated OPN levels can be associated with tumour recovery after radiotherapy, while OPN knockdown causes weak radiosensitisation [35]. Remarkably, Chang et al., reported that the induction of autophagy in human lung cancer cells, through exogenous beclin-1 (BECN1) overexpression, can result in decreased OPN levels as well as inhibition of cell radioresistance, at early stages of irradiation. It is suggested that autophagy plays dual roles in drug-resistance serving as a dynamic system. The pro-death or pro-survival roles of OPN-induced autophagy are highly dependent on the tumour type, treatment characteristics and tumourigenesis stages.

### 3.2. OPN and Drug Efflux of Cancer Cells

As one of the largest superfamilies of membrane proteins among both eukaryotes and prokaryotes, the ATP-binding cassette (ABC) transporters are widely expressed in physiological barriers and excretory tissues. These efflux pumps normally use the energy from ATP hydrolysis to drive the transport of various substrates across the cell membranes for protecting cells from foreign chemicals and therefore play an important role in cellular defence [36,37]. In addition to their profound physiological functions, various ABC transporters are implicated in tumour cell resistance to anticancer therapy, particularly, p-glycoprotein (P-gp; ABCB1, ATP-binding cassette sub-family B member 1), ATP binding cassette subfamily C member 1 (ABCC1), ABCC2, ABCG2 (also known as BCRP, breast cancer resistance protein), and the lung resistance protein. The overexpression of ABC transporters can efficiently pump out anticancer agents from cells and thus decrease drug intracellular effects, leading to multidrug resistance (MDR) in tumour cells [38,39,40,41].

It has been demonstrated that long-term treatment of daunomycin (DUN; daunorubicin) to PC3 prostate tumour cells, significantly increases OPN expression at both mRNA and protein levels in a concentration-dependent manner. Binding of secreted OPN to integrin αvβ3 and downstream focal adhesion kinase (FAK) activation markedly upregulates P-gp expression, leading to the promotion of DUN pumping-out activity. Further investigation of in vivo xenograft models also shows that OPN knockdown inhibits the P-gp expression and potentiates the cytotoxic effect of DUN [42]. Moreover, a detailed study of OPN function in melanoma revealed that stromal OPN enhances ABCG2 expression through the ERK2 dependent signalling pathway and selectively enriches side population (SP) phenotype, in B16F10 cells. The SP cells derived from tumour have high efflux capacity for Mitoxantrone and significant metastatic ability into lung and liver in OPN wild type (OPN+/+) mice [43]. Taken together, these data suggest that OPN could induce drug resistance, via the upregulation of ABC transporters, and may be a potential target for the inhibition of chemoresistance.

### 3.3. Epigenetic Changes

Epigenetics commonly refers to the inherited alteration of gene expression without changes in DNA sequence. Epigenetic changes due to DNA methylation, histone modification, and noncoding RNAs are extensively involved in mammalian development and human diseases, including cancer [44,45]. Recently, the growing body of evidence suggests that epigenetic alterations are one of the significant mechanisms of anticancer drug resistance [46,47,48].

It was reported that methylation and epigenetic silencing, in the promoter regions of a variety of tumour suppressor genes (TSGs), results in development of drug resistance in cancer cells. In both in vitro and in vivo studies, in hepatocellular carcinoma (HCC), the cancer stem cell-like CD133+/CD44+ cells with high OPN levels were more sensitive to DNA methylation inhibitor, 5 Azacytidine (5 Aza). Knockdown of OPN inhibits the expression of DNA (cytosine-5)-methyltransferase 1 (DNMT1), a key enzyme of DNA methylation. OPN-DNMT1 axis induces aberrant DNA methylation and inhibits sphere formation and migration of CD133+/CD44+ cells. These implicate OPN as a promising target for HCC resistance by methylome reprogramming [49].

Besides chromosomal modifications, non-coding RNAs, such as microRNAs(miRNAs), also play an important role in OPN-mediated chemoresistance. MiRNAs are noncoding single-stranded small RNAs at 19–25 nucleotides in length, which negatively regulate their target genes’ expression at the post-transcriptional level, by complementary binding to target mRNA 3′untranslated regions (UTRs), thereby participating in the regulation of malignant tumour behaviour such as tumourigenesis, proliferation, invasion, metastasis, and chemotherapy resistance [50,51,52]. The association between miRNAs and OPN in drug resistance of breast cancer has been investigated. For instance, Shevde et al., showed that the OPN-targeting miRNA hsa-mir-299–5p, is commonly downregulated in the breast cancer- spheroid-forming sub-population of cells (SFCs), with inherent drug resistance and enhanced tumourigenic potential. This results in a reversely high expression of secreted OPN by the SFCs, promoting tumourigenicity and angiogenesis [53]. In addition, Han et al., demonstrated that OPN is significantly overexpressed in MCF-7/ADR cells (the breast cancer cell line MCF-7 which developed resistance to Adriamycin) than in the wild-type MCF-7 cells. Induced overexpression of miR-181c inversely regulates OPN by direct targeting of OPN and reverses resistance of MCF-7/ADR cells both in vitro and in vivo. Clinically, miR-181c expression is negatively correlated with OPN expression, positively with chemosensitivity to ADR and overall survival of breast cancer patients. Thus, the miR-181c/OPN axis might be a potential therapeutic target for breast cancer patients, with acquired resistance to chemotherapy [54]. Furthermore, it has been demonstrated in cervical cancer cells, that overexpression of miR-181a plays a role in targeted inhibition of OPN expression in cisplatin (DDP)-resistant CaSki/DDP cells, which significantly promotes cell apoptosis, restrains cell proliferation, and increases cell sensitivity to DDP [55].

The above studies provide promising results in resensitising both cancer cell lines and animal models to chemotherapy through the epigenetic alterations affecting OPN, but further study is necessary to develop personalised treatment modalities for overcoming drug resistance in cancer patients.

### 3.4. OPN Is a Prime Regulator of EMT Process

Epithelial–mesenchymal transition (EMT) represents the phenotypic conversion from epithelial to mesenchymal cells, in which epithelial cells lose their cell identity and acquire mesenchymal characteristics [56]. EMT is normally observed during physiological conditions, whereas the same process can be executed by tumour cells during cancer development [57,58]. Recently, increasing evidence has indicated that pathological hyperactivated EMT is closely associated with elevated therapeutic resistance of cancer cells, which may be caused by aberrant activation of several key signalling pathways that promote EMT phenotype, including transforming growth factor beta (TGF-β), Notch, Wnt, and Hedgehog (Hh) [59,60,61].

Indeed, OPN has also been known as a critical regulator of EMT [62]. The possible mechanisms relating OPN-mediated EMT to cancer resistance are well-described in several studies. Using human breast cancer cell lines, Shevde et al., showed that OPN promoted the expression of mesenchymal markers N-cadherin, vimentin, Twist, Slug, and matrix metalloproteinase 9 (MMP9), by initiating nonclassical activation of the GLI-mediated Hh signalling pathway. In addition, OPN enhanced the expression of ABCB1 and ABCG2 proteins in a GLI-dependent manner. Collectively, epithelial–mesenchymal plasticity and drug efflux notably decreased the therapeutic effects of chemotherapeutic agents, including doxorubicin (DOX), paclitaxel, and cisplatin, that is, induction of MDR. Further, silencing OPN or GLI1 improved the susceptibility of breast cancer cells to all three cytotoxic chemotherapeutics [63]. Current evidence linking OPN to EMT-driven drug resistance was further supported by Zhang et al. [64]. This group showed that OPN was significantly up-regulated in H1975-AR and H1650-AR cells, which are EGFR-mutant NSCLC cells with the characteristic of acquired resistance to afatinib. Further analysis revealed that OPN overexpression promoted EMT phenotype by repressing expression of E-cadherin and upregulating N-cadherin and Vimentin. Conversely, silencing of OPN significantly suppressed EMT progression, and re-sensitise cells to afatinib. These results indicated that OPN might be involved in acquired resistance of H1650-AR and H1975-AR cells to afatinib.

These results suggest an additional possible mechanism relating OPN-mediated EMT to cancer drug resistance. Combination with OPN inhibitors may be a promising therapeutic option for cancer patients who develop acquired resistance to chemotherapy or targeted therapy drugs.

### 3.5. OPN Critically Regulates the Glycolytic Process

Aerobic glycolysis, also known as “Warburg effect”, produces abundant ATP for cancer cell survival and rapid proliferation that is considered the central component of metabolic reprogramming in cancer cells [65,66]. Increasing evidence suggests that dysfunctional glycolysis, caused by aberrant expression of glycolysis-related enzymes, could also play a significant role in tumour therapy resistance. For example, inhibition of pyruvate kinase isoform 2 (PKM2), a key glycolytic enzyme, has been reported to improve drug sensitivity in advanced breast, lung and colorectal cancer cells [67]. Similarly, 3-bromopyruvate (3-BrPA), an inhibitor of several glycolytic enzymes [68], showed a synergistic effect with DOX, sensitising DOX-resistant KG-1 leukaemia and RPMI8226 myeloma cells [69].

More recently, data have emerged indicating that OPN is a critical glycolytic regulator and could participate in drug resistance acquisition processes. For instance, Lu CY and her colleagues showed that OPN promotes glucose uptake and lactate production of HCC-LM3 cells, by modulating αvβ3-NF-κB signalling. Targeting OPN is sufficient to suppress HCC cell glycolytic activity and proliferation [70]. Additionally, OPN expression is significantly associated with platinum-based chemosensitivity in NSCLC patients. Ouyang et al., showed a relationship between OPN expression and patient’s individual sensitivity to platinum, and proposed that overexpression of OPN affects the regulation of lactate dehydrogenase A (LDHA), which is a key enzyme in the glycolytic pathway and, therefore, lactate production. LDHA upregulation and the acidic tumour microenvironment could promote cisplatin resistance and invasion of NSCLC cells [14]. Thus, glycolytic alterations, induced by targeting OPN, should be a promising therapeutic potential for cancer patients with drug-resistance.

## 4. OPN Mediates Signalling Pathways in Cancer Resistance

A number of recent studies have shown that OPN mediated signalling pathways participate in cancer drug resistance, through interaction with cell surface receptors, including integrin and CD44 cell receptors [71]. Here, we provide an overview of pre-clinical studies of OPN-induced major signalling, conferring resistant to many cancer drugs.

### 4.1. PI3K/Akt Signalling

The phosphoinositide 3-kinase (PI3K)/AKT signalling pathway is one of the most extensively studied and has been demonstrated to be a critical mechanism of drug resistance induced by OPN in cancer cells, e.g., NSCLC cells show less sensitivity to DDP, due to overexpression of OPN, and the OPN-induced drug resistance may be mediated by activation of the PI3K/ extracellular signal-regulated kinases (ERK) pathway [72]. Moreover, it has been found that OPN functions as a chemoresistant gene in HCC cells, which involves abnormal stimulation of the PI3K/AKT signalling pathway through both CD44 and integrin αvβ3. Blockage of the OPN pathway could reverse chemoresistance of DDP in HCC [73]. In acute myeloid leukaemia (AML), it is shown that resistance of CD34+ AML cells to daunorubicin (DNR) may be due to OPN mRNA overexpression. Targeting of OPN could decrease the mRNA levels of AKT, mammalian target of rapamycin (mTOR) and β-catenin gene axis and participate in the protection of cells against DNR [74]. In addition, Yi et al., give a novel mechanistic basis for OPN influence on sorafenib sensitivity in AML, especially in Fms-like tyrosine kinase-3 internal tandem duplication (FLT3-ITD) mutated AML. Their findings demonstrated that binding of FLT3-ITD mutated AML cells to OPN, through integrin αvβ3, could maintain cell survival and induce sorafenib insensitivity by activating the PI3K/Akt/glycogen synthase kinase (GSK) 3β/β-catenin signalling pathway [75]. Taken together, OPN/PI3K/Akt signalling influences drug sensitivity in several cancers, and this pathway may be the critical target for therapeutic design.

### 4.2. MAPK Signalling

Besides PI3K/Akt signalling, dysregulation of mitogen-activated protein kinase (MAPK) signalling, mediated by OPN, also shows a major role in cancer-acquired resistance to both targeted and chemotherapeutic drugs. It has recently been found that overexpression of OPN sufficiently reduces the efficacy of cetuximab (CTX) in NSCLC cells, by upregulating MAPK pathway-related proteins phosphorylated mitogen-activated protein kinase (p-MEK) and p-ERK, so presumably, OPN may confer resistance to CTX via activating the MAPK signalling pathway [76]. In addition, in the human gastric cancer cell line, SGC7901, Zhang et al. observed that lysophosphatidic acid (LPA)-induced overexpression of OPN is indispensable in the protective effect against Taxol-induced apoptosis, and mechanistic investigation found that Akt and MAPK/ERK signalling pathways are involved in the process [77].

### 4.3. EGFR Signalling

Inappropriate activation of the epidermal growth factor receptor (EGFR) and downstream signalling pathways have been implicated in the pathogenesis of various malignancies such as cell growth, invasion, adhesion, and migration [78]. Moreover, aberrant EGFR signalling, activated via mutation and amplification of EGFR, or phosphorylation of EGFR tyrosine kinase, is increasingly recognised as a driver of resistance to therapies in tumours [79,80,81]. It has been suggested that, in addition to ligand-induced signalling, the EGFR can be activated by the ligation of integrin, or CD44 with OPN, and is involved in acquired resistance. Lamour and collaborators showed that OPN expression is a feature of the glioma initiating cells (GICs), which is a phenotype of glioblastoma (GBM), and is implicated in progression, therapeutic resistance and recurrence [82]. OPN-CD44 interaction and the subsequent crosstalk between CD44 receptor and EGFR induced PI3K/Akt/mTOR pathway activation, presents a highly aggressive stem-cell like phenotype in GICs, to favour the sphere-growing capacity and tumourigenicity.

Besides the pathways mentioned above, other signalling pathways mediated by OPN also play a role in drug resistance. For example, it is shown that up-regulation of OPN and integrin αVβ3 contributes to acquired resistance to gefitinib, by activating the downstream FAK/Akt/Erk pathway in NSCLC cells in vitro [83]. While in U251 human neuronal glioma astrocytoma cells, recent data have reported that overexpression of OPN is involved in chemoresistance, via stimulating the NF-κB/B cell lymphoma 2 (Bcl-2) pathway. Specific inhibition of OPN can enhance the sensitivity of U251 cells to DDP and temozolomide (TMZ), by suppressing NF-κB activation and Bcl-2 expression [84]. In addition, it is found that OPN, as the Wnt target gene, is overexpressed in S1M cells, a mouse diffuse-type gastric cancer (DGC) cell line exhibiting cancer stem cell (CSC)-like feature. OPN contributes to DGC stemness and chemoresistance by promoting tumour sphere formation and induction of Bcl-xL, a pro-survival Bcl-2 family protein known to promote CSC survival. Inhibition of OPN in S1M results in greater sensitivity to DDP in vitro [85].

Taken together, the data from the current study indicate that targeting OPN mediated signalling pathways may be an effective therapeutic approach for circumventing resistance to conventional therapeutics in multiple cancers. However, the interaction between cancer cells and the microenvironment and the mechanisms of drug resistance are rather complicated. Consequently, additional investigations are necessary to better understand the roles of the OPN mediated pathways in anticancer drug resistance.

## 5. OPN Alternative Splicing in Cancer Drug Resistance

Alternative splicing (AS) is a rigorously regulated process via which a single precursor RNA (pre-RNA) is transcribed into different mature RNAs, contributing to the diversity of the proteome and cellular function. It is currently established that AS events of many genes, that can affect drug resistance, alter cancer cells’ sensitivity to various treatments, by changing the expression profile of their protein isoforms [86,87,88].

It is known that the OPN primary transcript is subject to alternative splicing, generating three main splicing isoforms, termed OPN-a (consists of all exons), OPN-b (which lacks exon 5), and OPN-c (which lacks exon 4) [89]. Recent studies have demonstrated that OPN splice variants in malignancies may have functional heterogeneity on drug affinity, eventually leading to drug resistance. For example, Mirzaei and colleagues reported that acquired up-regulation of OPN-b and OPN-c in CD34+ KG-1 (a human leukemic stem-like cell line) cells, as well as OPN-c in CD34- U937 (a sensitive AML cell line) cells, appears concurrent with enhancement of AKT/vascular endothelial growth factor (VEGF)/CXC chemokine receptor 4 (CXCR4)/signal transducer and activator of transcription 3 (STAT3)/interleukin-6 (IL-6) gene expression, which probably contributes to impede conventional chemotherapy (DNR/IDA and Ara-C in combination) drug-induced cell apoptosis and angiogenesis, ultimately resulting in chemoresistance [90]. Consistent with this, their previous studies revealed that OPN-b and OPN-c probably combat anti-angiogenesis effects of curcumin (CUR), with conventional AML regimen, via induction of the AKT/VEGF/STAT3/CXCR4/IL-6 molecular pathway [91]. Moreover, it is found that PC3 prostate cancer cells overexpressing OPN-b or OPN-c show higher viability in response to docetaxel (DXT)-induced cell death, compared to cells overexpressing OPN-a and empty vector controls. Further data indicate that the EMT program may be involved in modulating OPN-b or OPN-c resistance and pro-survival roles in DXT-treated cells [92]. It was also observed that OPN-c is upregulated in ACRP chemoresistant cells. Alterations in the resistance-associated EMT phenotype and P-gp gene expression, in response to OPN-c downregulation, reversed the sensitivity of ACRP ovarian cancer cells to DDP and DOX [93]. Similarly, OPN-c was preferentially increased and displayed a most significant effect, to promote colon cancer cell survival, following short 5-FU exposure, than other OPN splicing isoforms [94]. Further study on mechanisms suggested that the splicing of OPN-c, under 5-FU treatment, is largely controlled by the epigenetic factor Methyl-CpG binding protein 2 (MeCP2). MeCP2 phosphorylation on serine 421 reduces its binding to OPN exon 4, which could be regulated by nuclear calcium signalling and DNA methylation. However, inconsistent with these results, Yosifov et al., observed that OPN-a or OPN-b overexpressing multiple myeloma cells, have lower sensitivity to erufosine compared to the original cells, especially the OPN-a overexpressing variant [95].

Taken together, these results are in support of the understanding that overexpression of specific OPN variants are seemed to differentially contribute to tumour drug resistance, especially OPN-c. However, the mechanism is still poorly understood.

## 6. Future Prospective

For recent decades, cancer drug resistance has always been one of the most challenging topics in cancer biology. This article discussed the vital role OPN plays in anti-cancer drug resistance. Numerous studies have been in support of the recently developed understanding that OPN, including its alternative splicing isoforms, could be effective indicators of cancer drug resistance and might be of importance as potential targets for the improvement of oncotherapy.

### 6.1. Questions to Be Addressed in Future Studies

Although the role of OPN in promoting drug resistance is generally accepted, there are still several important issues that need to be further addressed. 

Firstly, due to the heterogeneity of tumour cells and the diversity of anticancer drugs, OPN has different regulatory effects on drug resistance, in different types of tumours (Table 3). Therefore, it is necessary to further and extensively confirm the mechanisms of OPN regulating cellular drug resistance for clinical development. Meanwhile, the reasons behind the distinctive roles of the OPN splicing variants in cancer drug resistance also need in depth mechanistic investigation. 

Secondly, most of the studies which have demonstrated findings associated with resistance to OPN are mainly cell culture studies in vitro, rather than in vivo or clinical studies (Table 3). Considering the complicated role of the tumour microenvironment on development of drug resistance, the contribution of OPN in the animal’s internal environment and the clinical model settings, might be of little importance compared with what is predicted by studies performed in in vitro cell culture models. 

Thirdly, potential confounding factors, relevant to detection of OPN protein, require attention. Because of expressive and functional heterogeneity, OPN splice variants require separate detection. The minor differences of the variants are now mainly distinguished by gene amplification technique. It is questionable whether immunohistochemistry is specific enough to determine the certain isoforms, as the antibody binding may be affected by the post-translational modifications of OPN [89,96,97]. It would be necessary to be individually detected in potential therapies. Currently, some methods have been considered promising for OPN quantification, in both research and clinical settings, and should be further studied in cancer patients. For example, Faria et al. described investigations involving an extended peptide internal standard, to track an unstable signature peptide for quantitative measurement of human OPN from plasma. This study used microflow liquid chromatography and tandem mass spectrometry (MFLC–MS/MS), and primarily validated the applicability of the method from 10 healthy individuals and 10 breast cancer patients [98]. By the similar immuno-mass spectrometry method, Macur et al., also successfully determined OPN concentrations in malignant and normal breast tissues, from six patients [99]. Additionally, a simple and label-free voltammetric aptasensor, using an RNA aptamer, previously reported to have affinity for human OPN, as the molecular recognition element, was developed for selective detection of OPN, in the presence of other interfering proteins (thrombin excepted), with reasonable detection and quantification limits [100]. Given AS, posttranslational modifications and forms of OPN, that could greatly contribute to the proteomic complexity, more studies incorporating precise methods of detection are needed to elucidate the predictive value of OPN in cancer drug resistance. 

### 6.2. OPN and Combination Therapies

Based on the fact that the predictive effect of OPN correlates with the treatment response, combination therapy targeting OPN and the above-mentioned oncogenic molecules or signalling pathways, may be a promoting option for overcoming cancer resistance. Different OPN inhibitors have been proposed to target OPN in vivo, such as parecoxib, brefelamide, and simvastatin. As a cyclo-oxygenase-2 inhibitor, parecoxinib was found to reduce OPN expression through blockade of the nuclear receptor subfamily 4 group A member 2 (NR4A2) and Wnt signalling pathways, in intestinal tumours of mice [101]. It has been suggested that brefelamide could exert an inhibitory effect on OPN expression and OPN-mediated cell invasion, through TGF-β/Smad signalling in A549 human lung carcinoma cells and might serve as a potential candidate for the targeted inhibition of OPN [102]. Simvastatin, (3-hydroxy- 3- methylglutaryl coenzyme A reductase inhibitor) as a natural OPN inhibitor, has been reported to inhibit cell proliferation by reducing OPN gene expression in AML cell lines [90]. Despite encouraging initial results with the different OPN inhibitors, the clinical benefit remains to be confirmed.

## 7. Conclusions

Here, we discuss aforementioned evidence of OPN and its variants’ potential in cancer drug resistance. Recent advances dramatically improve our understanding of OPN as a predictor for cancer recurrence following drug resistance. Activation of the OPN signalling could affect the therapy outcome. Although there is more to learn with regards to the mechanisms, it is clear that OPN is a promising biomarker to monitor cancer therapy resistance. Therapeutic targeting of OPN may be an efficient way to conquer treatment failure and significantly enhance antitumor activity. Accordingly, there is clinical prospect for pharmacological targeting of OPN, combined with other anticancer therapies, to overcome the obstacle of treatment of advanced cancers including chemotherapeutics, radio therapeutics as well as molecular targeted therapeutics towards resistance cancers, but more studies are necessary to confirm the clinical significance of OPN in cancer response to conventional therapy.

## Figures and Tables

**Table 3:** Summary of OPN and splice variants function on treatments response in different types of cancer cells.

Cancer Type	Preclinical Models	Biological Function	Reference
Lung cancer	In vitro	Exogenous beclin1-induced autophagy abrogated radioresistance of lung cancer cells by suppressing OPN.	[35]
	In vitro	OPN over-expression induced the expressions of EMT biomarkers, and promoted acquired resistance to afatinib in H1650-AR and H1975-AR cells.	[64]
	In vitro	OPN promoted cancer cell lactate and LDHA production and able to predict the response of A549 and SK-MES-1 to first-line platinum-based chemotherapy.	[14]
	In vitro	OPN enhanced cisplatin resistance of A549 lung cancer cells via stimulating the PI3K signalling pathway and upregulating ERCC1 expression	[72]
	In vitro	Overexpression of OPN reduced the sensitivity of NSCLC cells to cetuximab by upregulating MAPK pathway-related proteins.	[76]
	In vitro	OPN was overexpressed in acquired EGFR-TKI-resistant NSCLCs. OPN contributed to acquired gefitinib resistance by activating the integrin αVβ3/FAK pathway.	[83]
Breast cancer	In vivo	The OPN-targeting miRNA, hsa-mir-299–5p was commonly downregulated in the SFC subpopulations from three breast cancer cell lines, and the elevated OPN highlighted their role in tumour angiogenesis.	[53]
	In vitro	MiR-181c may regulate chemosensitivity by downregulating OPN, resulting in enhanced p53-dependent transactivation and apoptosis in resistant MCF-7 cells.	[54]
	In vitro	OPN enhanced EMT phenotype and the expression of ABCB1 and ABCG2 proteins in a GLI-dependent manner. Silencing OPN improved the susceptibility of breast cancer cells to DOX, paclitaxel and cisplatin.	[63]
Hepatocellular carcinoma (HCC)	In vitro	OPN expression was elevated during starvation-induced autophagy in HCCs. OPN engaged with integrin αvβ3 and sustained the stability of FoxO3a to induce autophagy, which further promoted stem-like phenotype of HCCs and resistance to epirubicin and cisplatin.	[33]
	In vitro and in vivo	OPN was closely related to the sensitivity of CD133+/CD44+ subgroup of HCC cells to 5 Aza.	[49]
	In vitro	OPN enhanced chemoresistance of cisplatin in HCC cells by activating PI3K/AKT signalling pathway.	[73]
	In vivo	OPN enhanced HCC glycolysis by activating the αvβ3-NF-κB signalling and hepatocarcinogenesis induced by DEN.	[70]
Gastric cancer	In vitro	Expression of OPN was mediated by the activation of Akt and MAPK/ERK pathways through the LPA2 receptor and protected SGC7901 cells from apoptosis induced by Taxol treatment.	[77]
	In vivo	OPN contributes to stemness of diffuse-type gastric cancer cells and chemoresistance by pro-moting tumour sphere formation and induction of Bcl-xL.	[85]
Colon cancer	In vitro	OPN-c could transmit the stress signal of cells upon 5-FU treatment in tumor microenvironment and promoted the survival of adjacent colon cancer cells.	[94]
Pancreatic cancer	In vitro	OPN/NF-κB signalling upregulated pancreatic CSC activity by activating autophagy.	[34]
	In vivo	Autophagy blockade sensitizes pancreatic CSCs to gemcitabine.	
Prostate cancer	In vitro	OPN upregulated P-gp expression through integrin αvβ3 in PC-3 cancer cells and inhibited DUN-Induced cell death. Knockdown of endogenous OPN enhanced the cytotoxicity of paclitaxel, doxorubicin, actinomycin-D, and rapamycin.	[42]
	In vivo	Knockdown of OPN enhanced the cytotoxicity of DUN in xenograft animal model.	
	In vitro	OPN-c or OPN-b overexpression in PC3 cells could mediate resistance and cell survival features in response to DXT-induced cell death.	[92]
Glioblastoma	In vivo	Endogenous OPN-mediated EGFR activation promoted GICs stemness phenotype and tumorigenicity.	[82]
	In vitro	OPN downregulation in U251 cells enhanced the effects of TMZ and DDP chemotherapy by targeting the NF-κB/Bcl-2 pathway.	[84]
Cervical cancer	In vitro	Overexpression of miR-181a can inhibit the expression of OPN and reduce DDP resistance in CaSki cells.	[55]
Ovarian cancer	In vitro	OPN-c was upregulated in cisplatin -resistant ACRP cells. Cells with OPN-c positive expression were more sensitive to cisplatin cytotoxicity compared with the negative control cells.	[93]
Melanoma	In vivo	OPN enhanced ABCG2 expression and enriched SP phenotype through ERK signalling in murine melanoma cells, and SP cells had high efflux capacity for mitoxantrone.	[43]
Acute myeloid leukemia (AML)	In vitro	Resistance of CD34+ AML cells to DNR might be relevant to increasing of OPN mRNA expression and activity of other mediators including AKT, mTOR, PTEN, and β-catenin.	[74]
	In vitro	Integrin αvβ3/ PI3K/ Akt/ GSK3β/ β-catenin/OPN axis was crucial for microenvironment mediated sorafenib insensitivity in FLT3ITD cells.	[75]
	In vitro	OPN isoforms, particularly b and c, alongside with VEGF isoforms and other gene pathways might promote chemoresistance in leukaemia.	[91]

## Data Availability

Not applicable.
